# Central macular choriocapillaris impairment as a manifestation of microvascular disease in eyes with subretinal drusenoid deposits

**DOI:** 10.1038/s41433-023-02654-1

**Published:** 2023-07-07

**Authors:** Solmaz Abdolrahimzadeh, Sandrine Anne Zweifel, Mariachiara Di Pippo, Anahita Bajka, Gianluca Scuderi, Andrew John Lotery

**Affiliations:** 1 Ophthalmology Unit, Neurosciences, Mental Health, and Sense Organs (NESMOS) Department, Faculty of Medicine and Psychology, University of Rome Sapienza, Rome, Italy; 2 St. Andrea Hospital, Via di Grottarossa 1035/1039, Rome, Italy; 3 Department of Ophthalmology, University Hospital Zurich (USZ), University of Zurich, Zurich, Switzerland; 4 Clinical and Experimental Sciences, Faculty of Medicine, University of Southampton, Southampton, UK

**Keywords:** Predictive markers, Prognostic markers

## Abstract

**Background/Objectives:**

Microvascular alterations and choroidal impairment are emerging as a pathologic pathway in age-related macular degeneration (AMD). This study aimed to evaluate the central macular choriocapillaris (CC) in eyes with subretinal drusenoid deposits (SDD) and the retinal microvasculature in patients with early AMD phenotypes.

**Subjects/Methods:**

This was an institutional, multicentric observational cross-sectional study. Ninety-nine eyes of 99 subjects; 33 eyes with SDD only, 33 eyes with conventional drusen (CD) only, and 33 eyes of healthy age-matched subjects were included. Comprehensive ophthalmologic examination and optical coherence tomography angiography (OCTA) was performed. The central macular flow area of the CC was analysed in the SDD group and the vessel density of the retinal superficial capillary plexus (SCP) and deep capillary plexus (DCP) was analysed in the SDD and CD groups using automated OCTA output parameters.

**Results:**

The flow area of the CC in the SDD group was significantly reduced (p ≤ 0.001) with respect to the healthy control group. There was a trend of reduction of vessel density of the SCP and the DCP in the SDD and CD group with respect to controls, although this did not reach statistical significance.

**Conclusions:**

OCTA data in the present report corroborate the role of vascular damage in early AMD with CC impairment in the central macular area in eyes with SDD.

## Introduction

Age-related macular degeneration (AMD) is a major cause of irreversible visual acuity loss in the population above 65 years of age [1]. The key pathological characteristic of early AMD is the presence of drusen that appear as yellow deposits of the retina on fundus examination. In the last three decades subretinal drusenoid deposits (SDD), or reticular pseudodrusen, have been recognized as an important phenotype in AMD. These deposits were first described by Mimoun et al. as localised in a peculiar yellowish pattern in the macular area with enhanced visibility on blue light [2]. The advent of advanced imaging technology such as near infrared reflectance (NIR) and spectral domain optical coherence tomography (SDOCT) have greatly enhanced our knowledge of retinal deposits in AMD [3]. SDD are well visible with NIR and are prevalently located in rod-rich macular areas, most frequently in and near the superior arcade, in the subretinal space between the photoreceptors and retinal pigment epithelium layer on SDOCT, whereas conventional drusen (CD), or so called soft drusen, are focal deposits of extracellular material beneath the retinal pigment epithelium [4–7].

SDD are associated with a higher risk of geographic atrophy and neovascularization in late AMD [7]. In vivo studies using enhanced depth imaging (EDI) SDOCT showed choroidal thickness alterations in eyes with SDD [8–10]. The choroidal vascularity index (CVI) is a measure of the luminal to the total choroidal area on Image J analysis of SDOCT scans. The CVI was successively adopted as a more reliable parameter to overcome bias linked to choroidal thickness measurement such as age, axial length, and systemic disease and was shown to be reduced in eyes with SDD [11–14].

Recently there have been a few studies with optical coherence tomography angiography (OCTA) to evaluate the microvascular parameters of the retina and choriocapillaris (CC) in eyes with SDD and CD [15–18], nevertheless, the mechanisms of SDD genesis still need to be clarified. Cicinelli et al. analysed 3 ×3 mm^2^ macular images with manual segmentation of the CC followed by image J analysis [17]. Alten et al. [15] and Nespar et al. [18] analysed automatically generated images prevalently in the outer superior sector (or outside) the Early Treatment Diabetic Retinopathy Study (ETDRS) grid, using Adobe Photoshop and a specifically designed image-processing algorithm, respectively. Interestingly, based on their findings, Alten et al. suggested that CC alterations in eyes with SDD are a generalised alteration of the macula rather than changes only in areas where SDD are localized [15]. Thus, we hypothesized that eyes with SDD could have central macular alterations irrespective of the site where deposits are visible on imaging.

The purpose of the present study was to evaluate microvasculature in early AMD phenotypes by evaluating the central macular CC flow area in eyes with SDD and the retinal vessel density in eyes with CD and SDD in comparison to healthy control subjects.

## Methods

Ninety-nine eyes of 99 subjects; 33 eyes with SDD, 33 eyes with CD, and 33 eyes of healthy age-matched subjects were included in this multicentric observational cross-sectional study. Patients and subjects were evaluated at the retina centre of the Ophthalmology Unit of the Sapienza University of Rome, St. Andrea Hospital and the Department of Ophthalmology of the University Hospital of Zurich. The Ethical Board of the Sapienza University of Rome and the Local Ethics Committee of the Canton of Zurich approved the investigation. Examinations were carried out in accordance with the principles of the declaration of Helsinki and all subjects gave written consent for study participation.

Inclusion criteria were diagnosis of early AMD in patients above 50 years of age with evidence of CD or SDD using NIR imaging and SDOCT in eyes without significant ocular media opacity. Exclusion criteria were eyes with neovascular membrane, signs of complete retinal pigment epithelium and outer retinal atrophy, evidence of epiretinal membrane, previous treatment with intravitreal anti-vascular endothelial growth factor agents, previous photodynamic therapy, more than 4 dioptres of spherical equivalent refractive error, intraocular pressure higher than 18 mmHg or glaucoma, diabetic or hypertensive retinopathy, neurologic pathologies or intraocular surgery in the 6 months prior to study enrolment. Healthy control subjects were selected from patients at the general ophthalmology unit who presented for routine ophthalmological examination and did not have the exclusion criteria of the study protocol or retinal and choroidal pathology.

Patients and subjects underwent comprehensive ophthalmologic examination including assessment of best corrected visual acuity and refractive error, slit lamp evaluation of the anterior segment, tonometry, fundus examination, photographic documentation of the posterior pole, fundus autofluorescence, and NIR imaging using the confocal scanning laser ophthalmoscope (Heidelberg Retina Angiograph, HRA2, Heidelberg Engineering, Heidelberg, Germany).

SDOCT raster images were acquired with the 17 parallel-lines of standard length and width according to the default settings of the Solix device (Solix, Optovue, Inc, Fremont, CA. Patients were included in the SDD group with evidence of a least 5 SDD in the diameter of a papillary disc area according to the classification system proposed by Zweifel et al. and Spaide et al. [4, 6]. In this group, the presence of even only one CD equal to or larger than 63 μm was a reason for exclusion. Patients were included in the CD group with at least one drusen larger than 125μm or 5 drusen between 63 and 125μm. One eye for each subject was included in the analysis; as the pathology considered in the study affects both eyes in a similar way [19]. If both eyes were eligible then one eye was selected at random according to the output value of a random number generator, assigning odd numbers to right eyes and even numbers to left eyes.

**Fig. 1:**
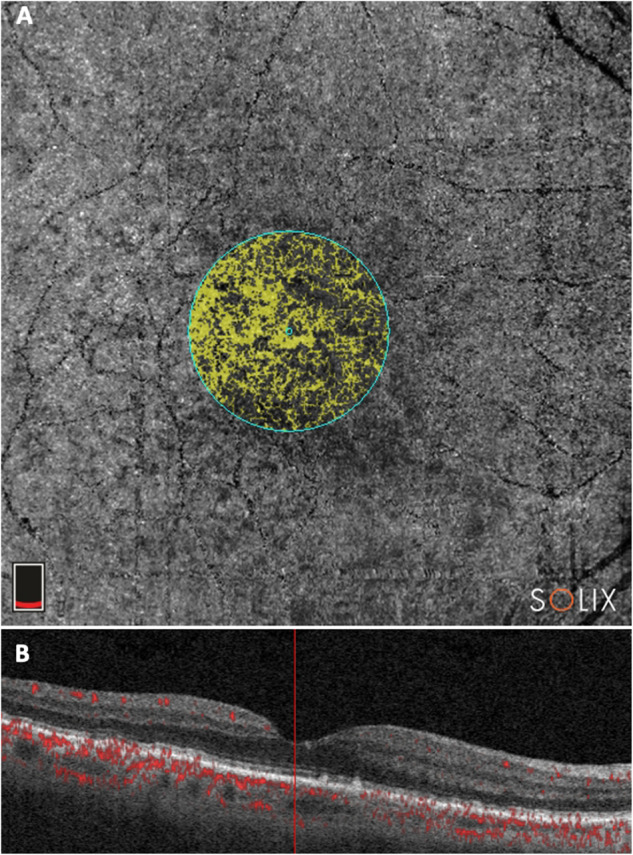
Choriocapillaris (CC) flow area on optical coherence tomography angiography in a patient with subretinal drusenoid deposits. **A** CC flow area is shown on an automated-generated circle centred on the fovea; **B** Cross-sectional scan of CC flow.

**Fig. 2:**
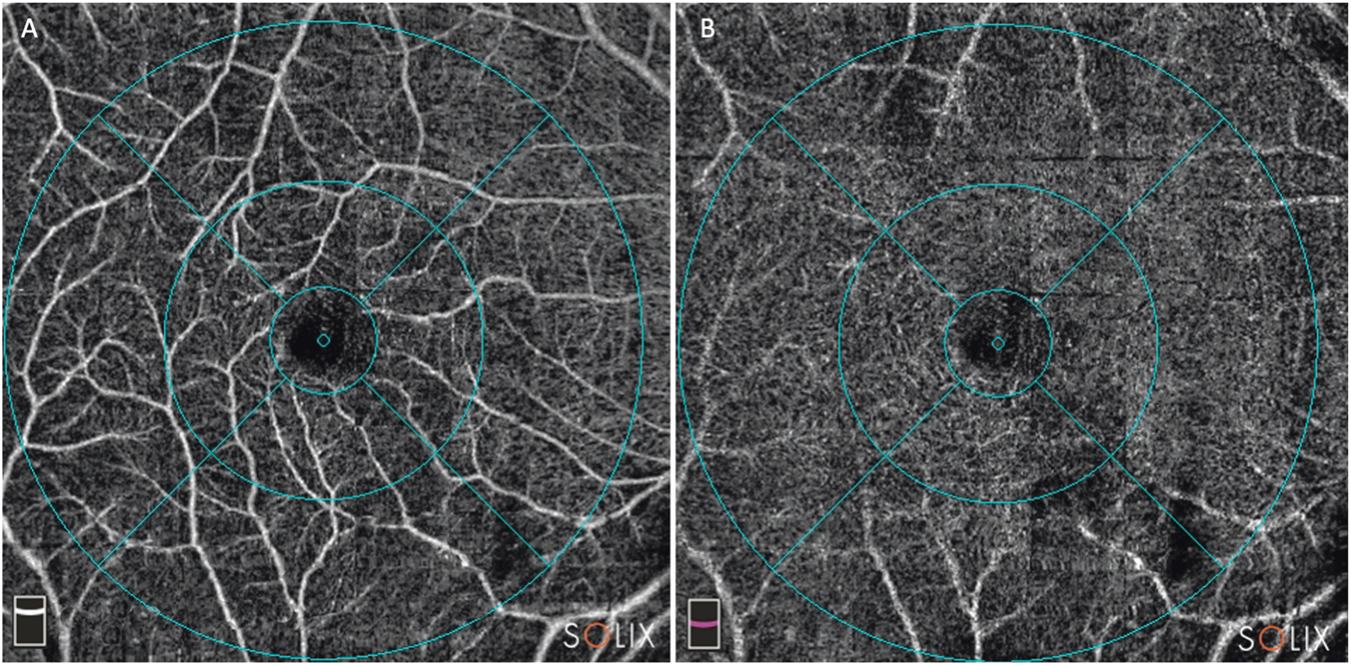
Superficial and deep capillary plexus (SCP, DCP) vessel density on optical coherence tomography-angiography HD AngioRetina 6.4 × 6.4 mm scan in a patient with subretinal drusenoid deposits. **A** SCP and **B** DCP with automatically superimposed overlay showing subfields.

OCTA evaluation was carried out with Solix AngioVue Retina® scans (6.4 mm × 6.4 mm) that through automated AngioAnalytics™ software enables the measurement of retinal vessel density (VD) and CC flow area. (Figs. 1 and 2) VD is the quantification of the proportion of pixels representing vessels out of total number of pixels for regions in the enface images of the superficial and deep capillary plexus (SCP and DCP) of the retina. The CC flow area measurement is made on an automatically generated 2mm^2^ diameter circle centred on the fovea detecting flow in a pre-defined CC slab. SDD do not produce shadow artifacts but owing to the challenging aspects of automated OCTA CC assessment caused by shadow artifacts in the presence of CD [15, 18], the analysis of the CC flow area was limited to the SDD and healthy control groups.

OCTA scans were checked for correct automated segmentation boundaries and the red flow signal in all study groups. The examination was repeated where motion artifacts were observed as dark lines on the enface angiograms. OCTA output images were compared with enface and cross-sectional images to identify any shadow artifacts on the CC. All examinations were performed by two experienced investigators (MDP, AB) and images were reviewed by senior investigators (SA, SAZ).

### Sample size justification and statistical analysis

The sample size was justified by the calculation performed on the detection of a difference between the VD parameter among the CD, SDD, and control groups using OCTA. We used the data extrapolated from a statistical analysis reported in a recent article, with OCTA analyses on the same type of patients [10], in order to estimate the sample size through balanced one-way analysis of variance power calculation on the means and standard deviations of the VD parameter relative to the three groups of patients analogous to those of our interest, setting power at 0.8 and significance level at 0.05. The following elements were considered in order to calculate the sample size: number of groups (equal to 3); between group variance (calculated as the variance between the means of the three groups, respectively equal to 0.390, 0.398, 0.406); within group variance (calculated on the basis of the standard deviation, equal to 0.020, which resulted to be identical in each of the three groups). In a one-way ANOVA study, sample sizes of 33, 33, and 33 were obtained from the 3 groups whose means were to be compared. The total sample of 99 subjects achieved 81% power to detect differences among the means versus the alternative of equal means using an F test with a 0.05 significance level. The size of the variation in the means was represented by their standard deviation, which was 0.01. The common standard deviation within a group was assumed to be 0.02.

Data were expressed as mean ± standard deviation or median and interquartile range for continuous variables, and the number of cases (and percentages) for categorical variables. All variables were tested for normality using the non-parametric Kolmogorov-Smirnov test. Continuous variables were compared by Student t-test for independent samples or by the analysis of variance (ANOVA), for normally distributed variables. A *p*-value ≤ 0.05 was considered statistically significant. A post hoc analysis was performed using a Bonferroni Test. Categorical variables were evaluated using the χ-square test or Fisher exact test when appropriate. Statistical analysis was performed using SPSS Software (version 28.0, SPSS INC, Chicago, Illinois, USA).

## Results

The mean age of the SDD group was 80.09 ± 6.94, the mean age of the CD group was 77.42 ± 7.42, and the mean age of the control group was 76.82 ± 7.91 years. The three groups were homogeneous and did not show statistically significant differences (*p*-value = 0.169). No difference in gender, best corrected visual acuity, and spherical equivalent between groups was found. Demographic data are shown in Table 1. Each patient group included eyes with SDD only or CD only assessed by NIR and SDOCT imaging.

**Table 1:** Demographic data of patients with subretinal drusenoid deposits (SDD), conventional drusen (CD), and control group.

	SDD (*n* = *33*)	CD (*n* = *33*)	*P*-value^*a*^	Control group (*n* = *33*)	*P*-value^*b*^
Age (Years)	80.09 ± 6.94	77.42 ± 7.42	0.446^c^	76.82 ± 7.91	0.169^e^
Sex (M/F)	17/16	13/20	0.322^d^	14/19	0.587^d^
BCVA (logMAR)	0.85 ± 0.12	0.82 ± 0.14	1.000^c^	0.86 ± 0.17	0.492^e^
Spherical Equivalent (Diopters)	−0.24 ± 1.23	0.29 ± 0.96	0.316^c^	−0.07 ± 1.70	0.252^e^

^a^Comparison of eyes with SDD versus eyes with CD.

^b^Comparison of eyes with SDD, eyes with CD, and control group eyes (control group).

^c^Post-hoc test with Bonferroni.

^d^Pearson’s chi square test, Bonferroni correction.

^e^ANOVA.

*BCVA* best-corrected visual acuity, *SDD* subretinal drusenoid deposits, *CD* conventional drusen.

OCTA image evaluation showed that the RPE and CC were correctly defined in all eyes by the built-in automated software of the OCTA device as assessed by the segmentation boundaries and the red flow signal on cross-sectional images. Shadow artifacts were not identified on OCTA angiograms in the SDD and healthy control groups when compared with enface and cross-sectional images.

The analysis of the difference between means showed a statistically significant reduction of the flow area of the CC in the SDD group with respect to the control group (*p* = 0.001). Covariance analysis of the OCTA parameters VD of the SCP and DCP between the SDD, CD, and control groups did not show statistically significant differences, even if there was a trend of reduction of VD of the SCP and DCP in the SDD and CD groups with respect to controls. All results are summarized in Table 2.

**Table 2:** Optical coherence tomography angiography data of patients with subretinal drusenoid deposits (SDD), conventional drusen (CD), and control group subjects.

	**SDD** (***n*** = ***33***)	**CD** (***n*** = ***33***)	***P-value***^*a*^	**Control group** (***n*** = ***33***)	***P-value***^*b*^	***P-value***^*e*^	***P-value***^*f*^
Deep capillary plexus vessel density (%)	44.95 ± 6.69	45.06 ± 6.48	1.000^c^	46.50 ± 4.01	0.500^d^	0.865^c^	1.000^c^
Superficial capillary plexus vessel density (%)	43.91 ± 4.14	44.37 ± 5.46	1.000^c^	46.37 ± 3.05	0.056^d^	0.070^c^	0.210^c^
	**SDD** (***n*** = ***33***)	**Control group** (***n*** = ***33***)	***P-value***^*e*^				
Choriocapillaris flow area (mm^2^)	1.71 ± 0.41	2.00 ± 0.26	0.001 ^g^*				

^a^Comparison of eyes with SDD vs. eyes with CD.

^b^Comparison of eyes with SDD, eyes with CD, and control group eyes.

^c^Post-hoc test with Bonferroni.

^d^ANOVA.

^e^Comparison of eyes with SDD and control group eyes.

^f^Comparison of eyes with CD and control group eyes.

^g^Student t-test.

*SDD* subretinal drusenoid deposits, *CD* conventional drusen.

*Statistically significant (*p* < 0.05).

## Discussion

In this study, we showed that the central macular CC flow area is reduced in early AMD in pure phenotypes with SDD compared to healthy control subjects.

In early AMD Pauleikhoff et al. reported prolonged choroidal filling using indocyanine green and fluorescein angiography [20]. Similarly, Smith et al. reported delayed filling of the CC using dye imaging in eyes with SDD and a histologic study reported patchy loss of the CC in one patient [21, 22]. Evidence from studies performed with imaging showed that choroidal thickness decreases with ageing and that CD and SDD are correlated to choroidal changes; while some authors reported choroidal thinning in eyes with SDD, other studies did not show significant thickness alterations [8–10, 23, 24]. This led to further investigations where the CVI was used as a more robust parameter and was reported to be reduced in eyes with AMD and lower in eyes with SDD with respect to those with CD [11, 12, 14].

OCTA is an in vivo imaging modality that is non-invasive as no dye is used and in-depth high-resolution images of the retinal SCP and DCP, and the CC are generated by acquiring repeated B-scans from every designated location where erythrocyte movement is detected [25]. Few studies have evaluated retinal and choroidal microvasculature alterations using OCTA in eyes with SDD or CD [15–18] and the role of the CC in SDD genesis is still not clear.

Chatziralli et al. designed a qualitative morphological study using OCTA and image J analysis and compared a group of eyes with SDD to a group of eyes with drusen less than 65μm. They found that eyes with SDD showed CC non-perfusion characterized by *absolute black* areas that they interpreted as almost total absence of perfusion of the CC or by areas of small focal *black holes*. Similar, but less intense, alterations were reported in the eyes with small drusen. In 3 patients with SDD they also reported *ghost-like vessels* that were not observed in eyes with drusen [26]. CC ghost vessels were reported in a histopathological study by Curcio et al. in areas with SDD [27]. Similarly, Mullins et al. found ghost vessels in eyes with soft drusen, possibly related to CC VD [28].

Cicinelli et al. used Image J software to analyse manually segmented CC and reported CC VD reduction in eyes with SDD, CD, and mixed phenotypes with respect to controls with no significant differences between eyes with SDD and CD [17]. Nespar et al. evaluated OCTA scans primarily in or outside the superior outer sectors of the ETDRS grid and evaluated the CC as *the percent choriocapillaris area of non-perfusion using a specific algorithm* and found a significantly larger extent of non-perfusion of the CC in eyes with SDD (with or without coexisting drusen) versus eyes with CD [18]. This discordance of results could be due to the variability in patient cohort analysis that were pure phenotypes in the study of Cicinelli et al. with respect to the mixed phenotypes (drusen and SDD) in the study by Nespar et al. [17, 18]. Nespar et al. [18] suggested the principal role of the choroid in the pathogenesis of SDD. This theory had previously been advanced by Alten et al. [15] who hypothesised that CC changes precede the development of SDD. Alten et al. selected images prevalently in the outer superior sector of the ETDRS grid and analysed automatically segmented image data using Adobe photoshop. These authors found reduction of the CC VD in eyes with SDD alone compared to healthy controls [15]. Interestingly, they found that CC alterations of the outer inferior sector, with less or no visible SDD lesions, were very similar to those of the outer superior sector where most lesions were observed. Thus, they suggested that CC alterations in SDD patients are a generalised alteration in the macula rather than a localised phenomenon, causing changes even in macular areas where SDD are not visible. Based on this rationale, we studied the CC vascularity using the automatically software-generated macular circle detecting flow in a pre-defined central CC slab of the Solix device. Similar to the results of Alten et al. and Nespar et al., who used specific algorithms to study peripheral macular areas where SDD were localized [15, 18], we found reduced CC flow area in the central macular area using automated OCTA output data, confirming the theory of generalized microvascular CC alterations in eyes with SDD. Montorio et al. analysed automated OCTA output data for all sections of the macular area and, similar to our results, reported reduced CC flow in eyes with SDD with respect to healthy controls [16]. However, these authors also reported that CC flow is reduced in eyes with CD versus controls. Although these results are in agreement with those of Cicinelli et al. who found CC VD reduction in eyes with both SDD and CD using image J elaboration of data, Montorio et al. did not account for possible shadow artifacts commonly caused by CD on automated OCTA outputs that can potentially lead to bias in CC flow area measurement [15, 18].

Montorio et al. also found a reduced VD of the macular SCP and DCP of the retina in an early AMD group versus controls although this did not reach statistical significance [16]. Our results are comparable as we found a similar trend, although not significant, in both the CD and the SDD groups. Interestingly, Toto et al. showed a trend of reduction of the VD of the SCP and DCP in early AMD, although not statistically significant [29]. Cicinelli et al. found that the VD of the SCP was reduced in patients with SDD (without outer retina atrophy) with respect to controls and reached statistical significance for the DCP [10]. This could perhaps be explained as image acquisition in this study was performed with a different OCTA device.

The pathophysiology of AMD is multifactorial and very complex involving genetic, oxidative, inflammatory, ischaemic, mitochondrial, and lipidic factors; choroidal impairment is emerging as an important pathological pathway [7]. The complement system is a mechanism of defence of the organism towards pathogens by activation of the membrane attack complex that can lyse pathogens but also damage cells [30]. Complement system components are also localised in the eye [31] and the domains around CC endothelial cells are reported as the principal sites of membrane attack complexes in the macula, thus, the site of the highest *complement-mediated stress* possibly indicating vascular injury and choroidal impairment as a pathological pathway in AMD [30–33]. The results using SDOCT imaging, CVI calculation, and OCTA in the present report, together with reports on histopathologic and dye technique outcomes corroborate the role of choroidal vascular alterations in the complex pathophysiological pathway of AMD with enhanced insight on the role of different deposits in the CD and SDD phenotypes. Furthermore, there seems to be a relationship between drusen and vascular loss in early AMD and as drusen load increases there is a loss of CC and increase in ghost vessels as shown by Mullins et al. in histologic and morphometric analysis [28].

Limitations in the evaluation of the CC using OCTA technology can be due to projection artifacts, shadowing artifact, and segmentation errors [34–36]. Projection artifacts occur when overlying retinal blood flow is detected in the choroid. This phenomenon is largely attenuated by the retinal pigment epithelium [34], and exclusion criteria in our study were retinal pigment epithelium and outer retinal atrophy and the OCTA software in our study included the 3D Projection Artifact Removal (PAR) algorithm. Nevertheless, drusen can cause signal attenuation or a shadowing effect that can cause artifacts in CC flow evaluation [34, 37]. Nespar et al. excluded regions with drusen from the analysis of their data to reduce the possible bias of signal attenuation and shadow artifacts, but their study protocol did not include the central macula [18]. Since our investigation was focused on the central macular area using automated OCTA output images, we excluded analysis of the CC in the CD group. Swept source OCTA technology seems to have less attenuation of signal [35]. CC evaluation can also be hindered by subtle segmentation inaccuracies largely owing to the variability of CC thickness across the macula [36].

Data comparison with studies in the literature is complicated owing to the use of different OCTA devices with variable offset limits for enface CC slabs [36]. A further limitation is possible bias in the comparison of results owing to the use of specific algorithms and Image J for elaboration of data [15, 17, 18] versus analysis of automated software outputs as in the present investigation [16].

In conclusion, we showed that the CC flow area is reduced in the central macular area in early AMD in pure phenotypes with SDD compared to healthy control subjects. CC flow area reduction of the central macular area in eyes with SDD alone, where deposits are rarely found, and the trend of reduced VD of the retinal SCP and DCP in SDD and CD phenotypes suggests generalised microvascular alterations in early AMD. This suggests a novel treatment for AMD might be revascularization of the CC. The qualitative and quantitative information provided by OCTA can contribute to improved classification and prognostic information on AMD phenotypes. However, future studies with further improved OCTA software to eliminate possible artifacts and provide uniform automated quantitative data on the CC are warranted.

## Summary

### What was known before:

- Microvascular alterations and choroidal impairment are emerging as a pathologic pathway in age-related macular degeneration. Conventional drusen and subretinal drusenoid deposits are correlated to choroidal changes.

### What this study adds:

- Choriocapillaris flow area is reduced in the central macular area in early age-related macular degeneration with subretinal drusenoid deposits as shown by optical coherence tomography angiography. Choriocapillaris impairment is linked to subretinal drusenoid deposits in early AMD suggesting that a novel treatment might be revascularization of the choriocapillaris.

## Data Availability

Data is available upon request.

## References

[1] Coleman HR, Chan CC, Ferris FL, Chew EY (2008). Age-related macular degeneration. Lancet.

[2] Mimoun G, Soubrane G, Coscas G (1990). Macular drusen. J Fr Ophtalmol.

[3] Zweifel SA, Spaide RF, Curcio CA, Malek G, Imamura Y (2010). Reticular pseudodrusen are subretinal drusenoid deposits. Ophthalmology.

[4] Zweifel SA, Imamura Y, Spaide TC, Fujiwara T, Spaide RF (2010). Prevalence and significance of subretinal drusenoid deposits (reticular pseudodrusen) in age-related macular degeneration. Ophthalmology.

[5] Spaide RF, Curcio CA, Zweifel SA (2010). Drusen, an old but new frontier. Retina.

[6] Spaide RF, Ooto S, Curcio CA (2018). Subretinal drusenoid deposits AKA pseudodrusen. Surv Ophthlmol.

[7] Sivaprasad S, Bird A, Nitiahpapand R, Nicholson L, Hykin P, Chatrizalli I (2016). Perspectives on reticular pseudodrusen in age-related macular degeneration. Surv Ophthalmol.

[8] Wu Z, Erica L, Fletcher C, Kumar H, Greferath UC, Robyn H (2022). Reticular pseudodrusen: a critical phenotype in age-related macular degeneration. Prog Ret Eye Res.

[9] Abdolrahimzadeh S, Parisi F, Marcelli M, Giustolisi R, Gharbiya M (2019). Optical coherence tomography evidence of macular ganglion cell-inner plexiform layer thinning in eyes with subretinal drusenoid deposits. Eye (Lond).

[10] Cicinelli MV, Rabiolo A, Sacconi E, Lamanna F, Querques L, Bandello F (2018). Retinal vascular alterations in reticular pseudodrusen with and without outer retinal atrophy assessed by optical coherence tomography angiography. Br J Ophthalmol.

[11] Corvi F, Souied EH, Capuano V, Costanzo E, Bennati L, Querques L (2017). Choroidal structure in eyes with drusen and reticular pseudodrusen determined by binarisation of optical coherence tomographic images. Br J Ophthalmol.

[12] Sacconi R, Vella G, Battista M, Borrelli E, Balasubramanian S, Querques L (2021). Choroidal vascularity index in different cohorts of dry age-related macular degeneration. Transl Vis Sci Technol.

[13] Viggiano P, Toto L, Ferro G, Evangelista F, Porreca A, Mastropasqua R (2022). Choroidal structural changes in different intermediate AMD patterns. Eur J Ophthalmol.

[14] Abdolrahimzadeh S, Di Pippo MC, Sordi E, Cusato M, Lotery AJ. Subretinal drusenoid deposits as a biomarker of age-related macular degeneration progression via reduction of the choroidal vascularity index: Eye (Lond). 2023;37:1365–70. 10.1038/s41433-022-02134-y PMC10169760 35739243

[15] Alten F, Heiduschka P, Clemens CR, Eter N (2016). Exploring choriocapillaris under reticular pseudodrusen using OCT-Angiography. Graefes Arch Clin Exp Ophthalmol.

[16] Montorio D, D’Andrea L, Mirto N, Cennamo G (2021). The role of optical coherence tomography angiography in reticular pseudodrusen. Photodiagnosis Photodyn Ther.

[17] Cicinelli MV, Rabiolo A, Marchese A, de Vitis L, Carnevali A, Querques L (2017). Choroid morphometric analysis in non-neovascular age-related macular degeneration by means of optical coherence tomography angiography. Br J Ophthalmol.

[18] Nesper PL, Soetikno BT, Fawzi AA (2017). Choriocapillaris non-perfusion is associated with poor visual acuity in eyes with reticular pseudodrusen. Am J Ophthalmol.

[19] Armstrong RA (2013). Statistical guidelines for the analysis of data obtained from one or both eyes. Ophthalmic Physiol Opt.

[20] Pauleikhoff D, Spital G, Radermacher M, Brumm GA, Lommatzsch A, Bird AC (1999). A fluorescein and indocyanine green angiographic study of choriocapillaris in age-related macular disease. Arch Ophthalmol.

[21] Smith RT, Sohrab MA, Busuioc M, Barile G (2009). Reticular macular disease. Am J Ophthalmol.

[22] Sarks J, Arnold J, Ho I-V, Sarks S, Killingsworth M (2011). Evolution of reticular pseudodrusen. Br J Ophthalmol.

[23] Alten F, Clemens CR, Heiduschka P, Eter N (2013). Localized reticular pseudodrusen and their topographic relation to choroidal watershed zones and changes in choroidal volumes. Invest Ophthalmol Vis Sci.

[24] Querques G, Querques L, Forte R, Massamba N, Coscas F, Souied EH (2012). Choroidal changes associated with reticular pseudodrusen. Invest Ophthalmol Vis Sci.

[25] Jia Y, Tan O, Tokayer J, Potsaid B, Wang Y, Liu JJ (2012). Split-spectrum amplitude decorrelation angiography with optical coherence tomography. Opt Express.

[26] Chatziralli I, Theodossiadis G, Panagiotidis D, Pousoulidi P, Theodossiadis P (2018). Choriocapillaris’ alterations in the presence of reticular pseudodrusen compared to drusen: study based on OCTA findings. Int Ophthalmol.

[27] Curcio CA, Messinger JD, Sloan KR, McGwin G, Medeiros NE, Spaide RF (2013). Subretinal drusenoid deposits in non-neovascular age-related macular degeneration: morphology, prevalence, topography, and biogenesis model. Retina.

[28] Mullins RE, Johnson MN, Faidley EA, Skeie JM, Huang J (2011). Choriocapillaris vascular dropout related to density of drusen in human eyes with early age-related macular degeneration. Invest Ophthalmol Vis Sci.

[29] Toto L, Borrelli E, Di Antonio L, Carpineto P, Mastropasqua R (2016). Retinal vascular plexuses' changes in dry age-related macular degeneration, evaluated by means of optical coherence tomography angiography. Retina.

[30] Mullins RF, Khanna A, Schoo DP, Tucker BA, Sohn EH, Drack AV (2014). Is age-related macular degeneration a microvascular disease?. Adv Exp Med Biol.

[31] Bhutto IA, Baba T, Merges C, Juriasinghani V, McLeod DS, Lutty GA (2011). C-reactive protein and complement factor H in aged human eyes and eyes with age-related macular degeneration. Br J Ophthalmol.

[32] Skeie JM, Fingert JH, Russell SR, Stone EM, Mullins RF (2010). Complement component C5a activates ICAM-1 expression on human choroidal endothelial cells. Invest Ophthalmol Vis Sci.

[33] Seth A, Cui J, To E, Kwee M, Matsubara J (2008). Complement-associated deposits in the human retina. Invest Ophthalmol Vis Sci.

[34] Borrelli E, Sarraf D, Freund KB, Sadda SR (2018). OCT angiography and the evaluation of the choroid and choroidal vascular disorders. Prog Ret Eye Res.

[35] Tiosano L, Corradetti G, Sadda SR (2021). Progression of choriocapillaris flow deficits in clinically stable intermediate age-related macular degeneration. Eye.

[36] Corvi F, Su L, Sadda SR (2021). Evaluation of the inner choroid using OCT angiography. Eye.

[37] Zhang Q, Shi Y, Zhou H, Gregori, Chu Z, Zheng (2018). Accurate estimation of choriocapillaris flow deficits beyond normal intercapillary spacing with swept source OCT angiography. Quant Imag Med Surg.

