# Correction: Central macular choriocapillaris impairment as a manifestation of microvascular disease in eyes with subretinal drusenoid deposits

**DOI:** 10.1038/s41433-023-02689-4

**Published:** 2023-08-08

**Authors:** Solmaz Abdolrahimzadeh, Sandrine Anne Zweifel, Mariachiara Di Pippo, Anahita Bajka, Gianluca Scuderi, Andrew John Lotery

**Affiliations:** 1 Ophthalmology Unit, Neurosciences, Mental Health, and Sense Organs (NESMOS) Department, Faculty of Medicine and Psychology, University of Rome Sapienza, Rome, Italy; 2 St. Andrea Hospital, Via di Grottarossa 1035/1039, Rome, Italy; 3 Department of Ophthalmology, University Hospital Zurich (USZ), University of Zurich, Zurich, Switzerland; 4 Clinical and Experimental Sciences, Faculty of Medicine, University of Southampton, Southampton, UK

**Keywords:** Predictive markers, Prognostic markers

Correction to: *Eye* 10.1038/s41433-023-02654-1, published online 07 July 2023

In the original article, the author name “Solmaz Abdolrahimzadeh” was given incorrectly as “Solmaz Abolrahimzadeh”. The original article has been corrected.

